# Accumulation Potential Cadmium and Lead by Sunflower (*Helianthus annuus* L.) under Citric and Glutaric Acid-Assisted Phytoextraction

**DOI:** 10.3390/ijerph20054107

**Published:** 2023-02-25

**Authors:** Zhixin Niu, Xiaojun Li, Mohammad Mahamood

**Affiliations:** 1 Department of Environment, Shenyang University, Shenyang 110021, China; 2 Institute of Applied Ecology, Chinese Academy of Sciences, Shenyang 110016, China; 3 Department of Biology, Deanship of Educational Services, Qassim University, Buraidah 52571, Saudi Arabia

**Keywords:** phytoextraction, sunflower, organic acids, lead, cadmium

## Abstract

Organic acid assistance is one of the effective methods for phytoremediation of heavy metal contaminated soil. In this experiment, the addition of citric and glutaric acids was selected to improve the accumulation of cadmium and lead by *Helianthus annuus* L. Results showed that citric and glutaric acids elevated the growth of the plants and stimulated Cd/Pb uptake by plant in single Cd/Pb treatments, but glutaric acid showed inhibitory action on the uptake of metals in complex treatments. Organic acids impacted the translocation of Cd/Pb differently, and citric acids (30 mg·L^−1^) enhanced the translocation of Cd to aerial parts of the plants in Cd (5 mg·kg^−1^) and Cd (10 mg·kg^−1^) plus Pb treatments. Glutaric acid (30 mg·L^−1^) could promote the translocation factors in the complex treatments of Cd (5 mg·kg^−1^) with Pb (50, 100 mg·kg^−1^) added. The application of citric and glutaric acid could be conducive to increase floral growth when proper doses are used, and incorporation of these organic acids can be a useful approach to assist cadmium and lead uptake by sunflower. However, growth, bioaccumulation, and translocation of metals may differ due to the metals’ property, kinds, and concentrations of organic acids.

## 1. Introduction

Growing industrialization with economic growth and rapid urban sprawl in recent years is deemed the most important factor for debasing the environment quality due to heavy metal pollution [1], such as by cadmium (Cd) and lead (Pb). Metallic elements are toxic to living organisms to a large extent [2]. The biotoxicity of Cd and Pb has been well proved for various plant species, where they severely cause nutrient deficiency resulting in undesirable effects on metabolic processes and stunted growth [3].

Until now, there have been a number of conventional technologies, including chemical and physical methods, which have been used for the maintenance of a clean environment [4]. Generally, the majority of these techniques are costly and environmentally destructive, despite high efficiency [5]. Phytoextraction is one of the phytoremediation techniques which is dependent upon the ability of plants to accumulate impurities, and to remove them from soil or water. It has been widely applied as an effective and eco-friendly alternative to expensive conventional methods [6,7]. 

In recent decades, sunflower (*Helianthus annuus* L.), as a plant of the family Asteraceae, has attracted much attention for use in phytoextraction, as it could be grown under stressful conditions, such as heavy metal stress [2,8,9,10,11]. Sunflower can accumulate relatively large amounts of metallic contaminants, besides translocating a good amount of the same from roots to shoots, and producing large quantities of biomass [4,12]. Willshire et al. [13] found that *Helianthus annuus* L. was a robust and suitable plant that could be used for extracting and repairing cadmium, lead, and zinc from soil under certain conditions. 

Moreover, the success of phytoextraction partially relies on the solubility and availability of metal in soil for root uptake. Usually, heavy metals adsorbed in soil particles are difficult to be integrated by plants [14,15,16]. Recently, some chemical amendments, including EDTA, limestone, etc., have been used to promote the phytoextraction process [17], but chemical amendments are toxic to plants and adverse to soil microorganisms which have significant impact on plant growth, even though these amendments can increase the efficiency of phytoextraction [18]. For enhancement of phytoextraction, a developmental alternative to chemical amendments is the utilization of chelating agents, such as low molecular mass organic acids (LMMOAs), which is a practical approach that has received widespread attention in phytoremediation [19,20,21]. The chelate-promoting potential of LMMOAs have been well documented for *Helianthus annuus* L., *Lemna minor* L., *Brassica napus* L., *Chrysopogon zizanioides*, and *Brassica juncea* L. under different heavy metals stress [11,22,23].

LMMOAs, including citric acid (CA), glutaric acid (GA), and oxalic acid, which are components of root exudates, can adjust redox coupling processes, anion-cation exchange, and respiration in soils [24]. The study of Najeeb et al. [25] reported that LMMOAs could promote the growth of plants by altering the speciation of elements and activating bioavailability of heavy metals, and thus uptake of essential ions by plants could be drastically modified. It has also been confirmed that LMMOAs could chelate with Cd and Pb, a process that can help to reduce the activity coefficient of free ions, thereby decreasing toxicity to plants and their own amount in the soil in order to realize remediation [2,10,11,26].

Although LMMOAs are excellent representatives of novel reagents, alleviating heavy metals-induced detrimental effects, and improving phytoextraction [27], relatively little literature is available on the amendment of citric acid and glutaric acid in the accumulation of Cd and Pb by sunflower. Therefore, the purpose of this paper is to explore the effects of two organic acids (OAs), namely, CA and GA, on the growth responses and the accumulation of metals by sunflower under single Cd or Pb and the complex of Cd and Pb stress. Our observations will provide scientific information for promoting the application of phytoremediation technology induced by chelates. 

## 2. Materials and Methods

### 2.1. Plant Preparation

Seeds of *Helianthus annuus* L. were obtained from Shenyang Agricultural University Seed Co., Ltd. Surface sterilizing of seeds was conducted by immersion in 20% *v*/*v* bleach, and seeds immersed in sterile water were shaken on an orbital shaker (Beijing) at 144 r/min for 6 h before being sown onto steel trays with medical gauze in an incubator; seeds with microbial growth were abandoned. Ten sterile seedlings which reached 6–7 cm approximately were transplanted to pots with 5.0 kg polluted soil and control soil, respectively. The duration of the culture was 45 days.

### 2.2. Experimental Design

Cadmium (CdCl_2_·2.5H_2_O) and lead (Pb(NO_3_)_2_·H_2_O of reagent grade were diluted separately in deionized water. Concentrations in this experiment were designed in line with the polluted soil in the Zhangshi Irrigation Area (SZIA) (E 103°42′45, N 26°38′57) in China, which is a typical area of heavy metal pollution caused by sewage irrigation over about 30 years. Solutions of different concentrations were added to the soil medium (organic matter 14.72 g·kg^−1^, CEC 159.70 mmol·kg^−1^, pH 6.80) and mixed evenly. The experimental treatments were as follows: (1) CK (no Cd, Pb); (2) Cd5, Cd10 (Cd 5, 10 mg·kg^−1^); (3) Pb50, Pb100 (Pb 50, 100 mg·kg^−1^) and (4) Cd5+Pb50, Cd10+Pb50, Cd5+Pb100, Cd10+Pb100 (Cd 5 mg·kg^−1^ + Pb 50 mg·kg^−1^, Cd 10 mg·kg^−1^ + Pb 50 mg·kg^−1^, Cd 5 mg·kg^−1^ + Pb 100 mg·kg^−1^, Cd 10 mg·kg^−1^ + Pb 100 mg·kg^−1^). Organic acids (citric and glutaric acid of reagent grade) were separately dissolved in deionized water to concentrations of 10 mg·L^−1^, 20 mg·L^−1^, and 30 mg·L^−1^. Solutions (500 mL) were added into the soil culture and homogenized for 1 day before the start of the experiment. Treatments were in triplicate for replication, and watered twice a week during the experiment.

### 2.3. Analysis of Biomass and Heavy Metals

Plant samples were collected every 15 days and the aerials and roots were separated by stainless blade. All parts were washed with detergent solution, followed by rinsing several times with distilled water. Plant samples, including aerials and roots, were dried for 72 h in an oven at 80 °C, and the weights of samples were recorded by an electronic balance (0.1 mg accuracy). Plant samples (0.5 g) were digested with 10 mL of concentrated HNO_3_ (trace pure, Inorganic Venture Co., Ltd., Beijing, China) at 105 °C for 30 min on a hot plate (Beijing). Then, the digestion volume was diluted to 20 mL with deionized water and all sample solutions were analyzed by flame atomic absorption spectroscopy (Spectra AA220, Varian). The absorbance values were measured at 228.8 nm and 283.3 nm using cadmium and lead hollow-cathode lamps as source of radiation, respectively, and an air-acetylene flame.

### 2.4. Quality Control and Statistical Analysis

Reagent blanks in this experiment were used to correct the analytical values, and all analyses were conducted in triplicate. Certified reference materials of cadmium and lead (SH-AA08N-1, SH-AA29N-1) were obtained from the National Institute of Metrology (Beijing, China) for quality assurance. The recovery rates for Cd and Pb ranged from 93.6% to 107.7% for both digestion and sequential extraction. 

The parameters, PRB (promotion rates of biomass) and TF (translocation of Cd or Pb) as defined in Equations (1) and (2), which were computed from the biomass of plant parts and accumulation of Cd and Pb in treatments, were used to discuss the results.

PRB (in single and complex treatments) = (Biomass of plants with CA or GA addition − biomass of plants without CA or GA addition)/biomass of plants without CA or GA addition (1)

TF = (Cd or Pb uptake by aerials)/(Cd or Pb uptake by roots)(2)

The ANOVA and Pearson correlation analyses on all data obtained in this experiment were performed by SPSS statistical software (SPSS Inc., Chicago, IL, USA). Differences at the level of *p* < 0.05 were considered statistically significant.

## 3. Results

### 3.1. Biomass Promotion in Citric Acid and Glutaric Acid Treatments

**Figure 1:**
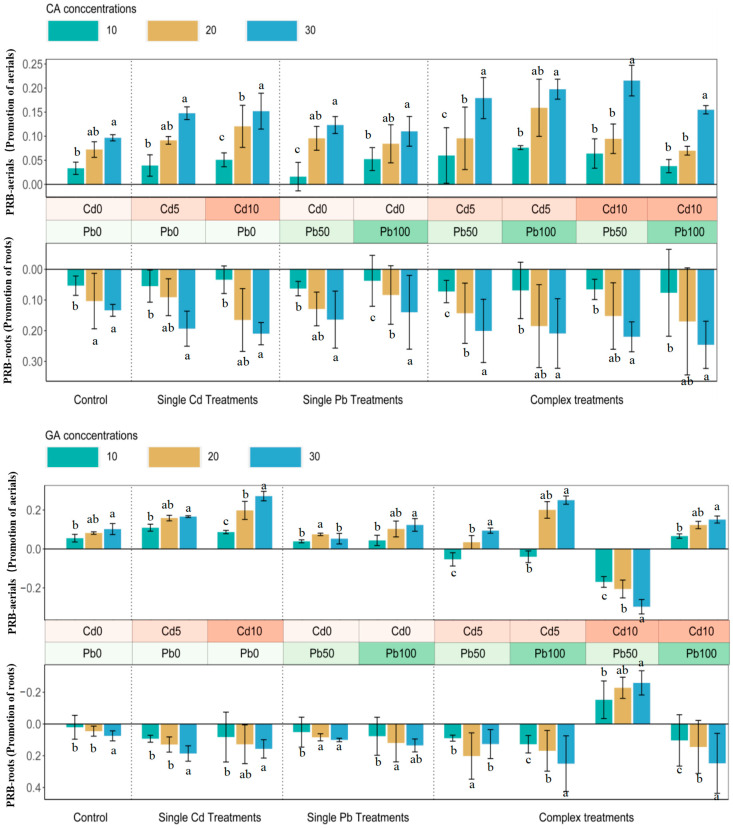
PRBs of aerials and roots of sunflower with citric acids and glutaric acids addition after 45 days. CA, citric acid; GA, glutaric acid; PRB, promotion ratio of biomass. Bars with different letters indicate significant difference at *p* < 0.05. Error bars represent ± S.E.

Citric acid improved the growth of sunflower, and the promotion showed an increasing tendency with the concentrations of CA in single and complex treatments. In single Cd10 and Pb50 treatments, the maximum shoot and root promotions were 20.97%, 15.21%, and 16.38%, 12.33% (*p* < 0.05), respectively (Figure 1). The promotion ratios in Cd treatments were more than those in Pb treatments, and PRBs decreased with increasing Pb concentrations. The highest PRB ratios in complex treatment appeared in Cd10+Pb50 (21.55%, aerials; 24.60%, roots) (*p* < 0.05). The Pb additions could enhance the biomass growth of aerial parts, and 50 mg·L^−1^ Pb increased the biomass considerably in Cd10 treatments.

The biomass elevations of roots and aerials in Cd treatments were higher than those in Pb treatments when glutaric acids were used, and the PBRs of sunflower increased with the GA concentrations (Figure 1). On the contrary with CA treatments, the additions of GA inhibited the aerials biomass in Cd10+Pb50, and GA reduced the growth of roots except for Cd10+Pb50.

### 3.2. The Bioaccumulation of Cadmium and Lead

**Figure 2:**
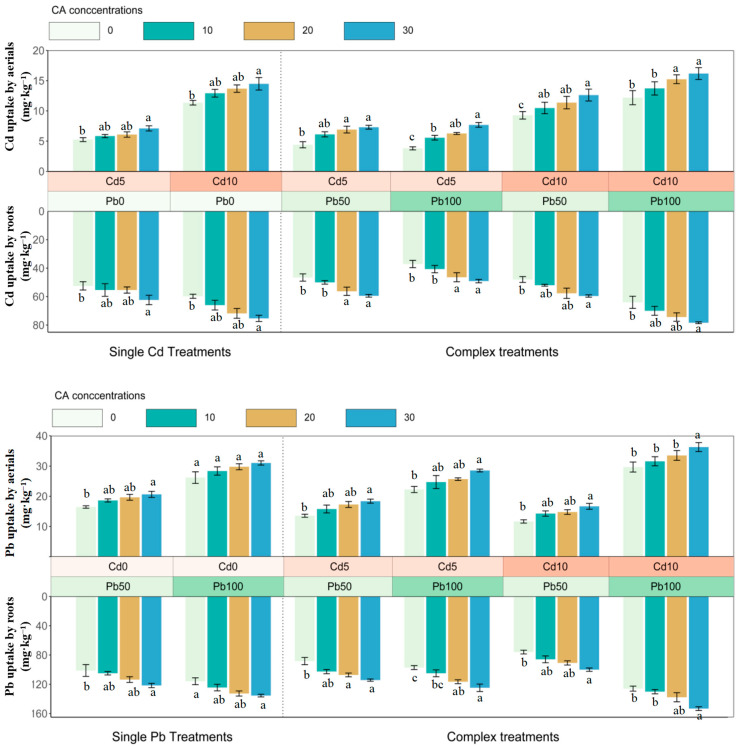
Cd and Pb accumulation in aerials and roots of sunflower in CA treatments after 45 days. CA, citric acid. Bars with different letters indicate significant difference at *p* < 0.05. Error bars represent ± SE.

**Figure 3:**
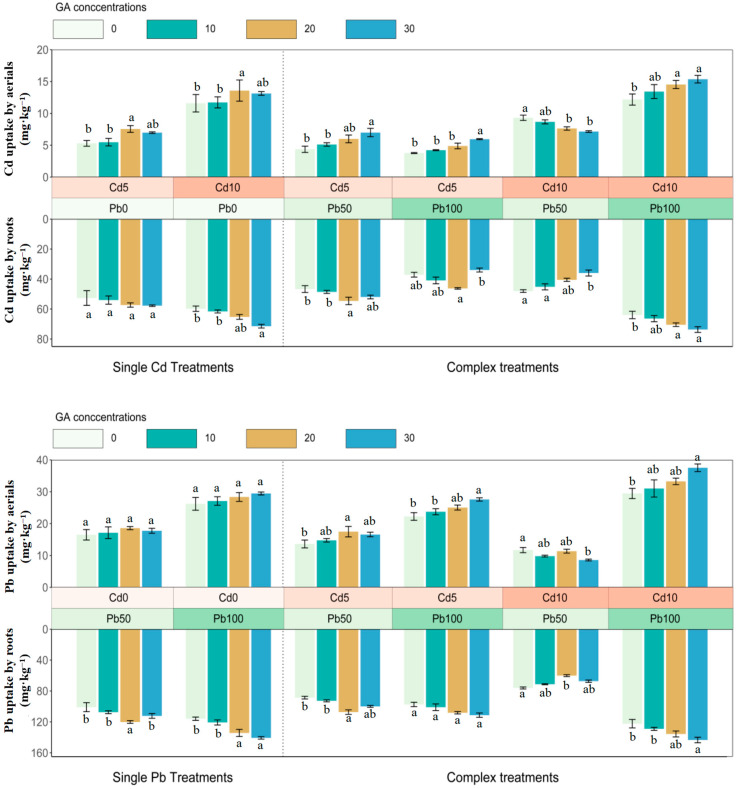
Cd and Pb accumulation in aerials and roots of sunflower in GA treatments after 45 days. GA, glutaric acid. Bars with different letters indicate significant difference at *p* < 0.05. Error bars represent ± SE.

In single treatments, citric acid elevated Cd or Pb enrichment in roots and aerials and the uptake increased with concentrations of heavy metals (Figure 2). The highest Cd and Pb accumulations appeared in Cd10 and Pb100, respectively, with 30 mg·L^−1^ CAs addition (Cd: 14.48 mg·kg^−1^, aerials; 75.32 mg·kg^−1^, roots; Pb: 31.06 mg·kg^−1^, aerials; 140.71 mg·kg^−1^, roots) (*p* < 0.05) (Figure 2). The increasing concentrations of glutaric acid could promote the Cd uptake by roots in Cd10 treatments, but the effect of glutaric acid on the Pb accumulation was not obvious except in Pb100 treatments (Figure 3).

In complex treatments, citric acid could improve the Cd and Pb uptake by roots and aerials, the highest Cd (78.40 mg·kg^−1^, root; 16.18 mg·kg^−1^, aerials) and Pb (153.28 mg·kg^−1^, root; 36.27 mg·kg^−1^, aerials) extracted by sunflower appeared in Cd10+Pb100 (*p* < 0.05) (Figure 2). The Cd uptake by aerials and roots showed a decreasing pattern with the increase in OA concentrations when glutaric acid was applied, and there was no improvement in Pb accumulation due to glutaric acid in Cd5+Pb50 and Cd10+Pb50 (Figure 3). Besides, the existence of Cd/Pb ions inhibited the accumulations of Pb/Cd, except for Cd10+Pb100 (Figure 2 and Figure 3).

### 3.3. The Translocation of Cadmium and Lead in Sunflower

**Figure 4:**
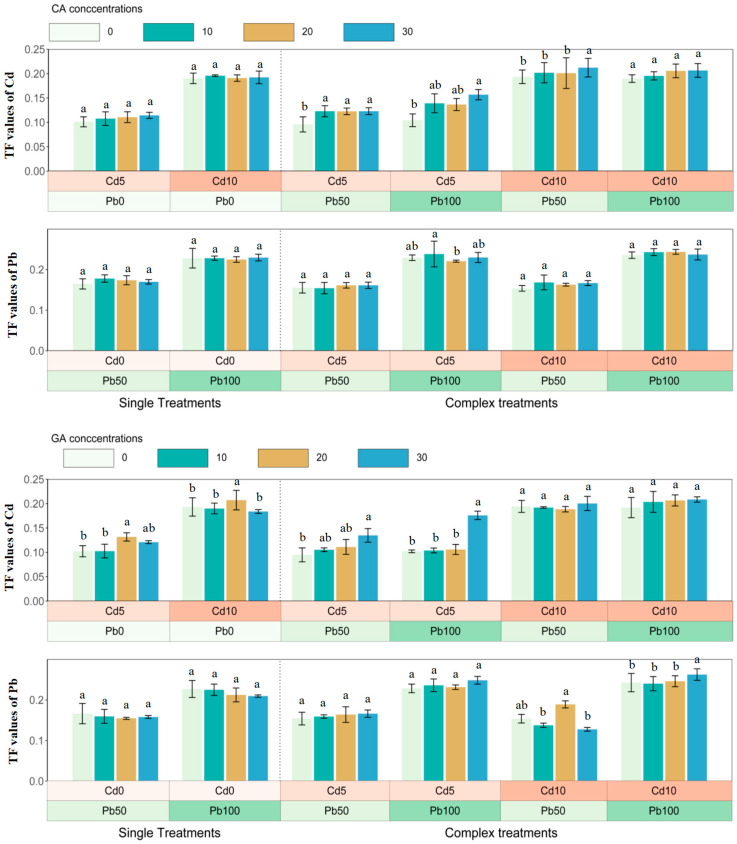
Translocation factors of Cd and Pb in sunflower with CA and GA addition, respectively, after 45 days. CA, citric acid; GA, glutaric acid; TF, transfer factor. Bars with different letters indicate significant difference at *p* < 0.05. Error bars represent ± SE.

The TF values increased together with the rise in concentrations of metals. However, the impacts owing to OA on the transfer of Cd and Pb were different in single treatments (Figure 4). In complex treatments, 30 mg·L^−1^ CAs improved TFs of Cd to a great extent in Cd5+Pb100 and Cd10+Pb100 treatments, and 100 mg·kg^−1^ Pb could promote the Cd transfer from roots to aerials. TF values of Cd also increased when the concentrations of glutaric acids were increased in Cd5+Pb50, and the highest value (0.20, 0.21) appeared in Cd10+Pb50 and Cd10+Pb100 treatments, respectively, when 30 mg·L^−1^ GAs was added (*p* < 0.05). TFs of Pb were boosted when Cd was added, and the maximum TFs were 0.25 and 0.26 in Cd5+Pb100 and Cd10+Pb100 when 30 mg·L^−1^ GAs was added (*p* < 0.05).

### 3.4. Relations between OAs Concentrations and PRB and Cd/Pb Uptake

**Figure 5:**
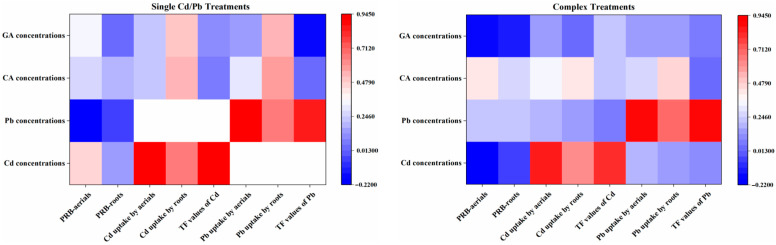
Correlation between concentrations of CA, GA, Cd/Pb and PRB, Cd/Pb uptake, TF of sunflower in single and complex treatments based on 180 (n) soil samples. CA, citric acid; GA, glutaric acid; PRB, promotion ratio of biomass; TF, transfer factor.

The metal uptake by roots in single treatments exhibited an increasing pattern when concentrations of Cd were increased. Pb uptake by roots also climbed with the increase in the concentrations of citric acid and glutaric acid (Figure 5). In complex treatments, the Cd/Pb accumulation by aerials and roots significantly increased the concentrations of Cd or Pb. The PRB values of aerials, Cd and Pb uptake by roots showed positive correlations with CA, but GA concentrations had no significant effect on PRB, Cd, and Pb uptake.

## 4. Discussion

### 4.1. Biomass Promotion in Citric Acid and Glutaric Acid Treatments

The application of OAs has been reported as stimulating growth in sunflower in much of the literature. Zaheer et al. [15] suggested that CA addition could ameliorate gaseous exchange properties, microstructure of chloroplast, and photosynthetic pigments, which related to overall improvement in plant growth. Under heavy metal stress condition, biomass promotion of plant might be due to the chelating role of CA. CA could enhance the absorption and utilization of essential nutrients through the formation of complex with nutrients, and it could induce metals chelation to reduce free metal ions in plants and maintain photosynthetic balance [28]. In this experiment, the PRB ratios of aerials declined with the increasing Pb concentrations when the CA were added in Pb treatments. Pb has been confirmed to influence the carotenoids and photosynthetic pigments content of plants. The addition of CAs could increase the mobility and bioavailability of lead. This process made the CAs-Pb chelate easy to directly contact with plant roots [4,29], and lead-organo entering into plants might interfere with organelle metabolism processes, eventually destroying the integrity of the cell membrane, resulting in a weakening of photosynthesis [30,31]. Meanwhile, Wang et al. [32] suggested that excessive chelating agent might remove divalent cations from the plasma membrane, which would result in the destruction of the physiological barrier of root systems, leading to irreversible impacts on the growth of plants [33]. In accordance with our findings, previous studies have reported that the combined applications of Cd and Pb could decrease plant growth more than single applications of Cd or Pb by inhibiting the entire metabolic activity and interrupting the mineral intake [19,34]. The addition of OAs tended to mitigate the toxic impact of heavy metals on plants by regulating normal functions and increasing nutrient intake. However, GA hindered the growth of sunflower in our experiment, probably due to the morphological and physiological characteristics of plants and the toxicity of metal-chelator complexes.

### 4.2. The Bioaccumulation of Cadmium and Lead

OAs are widely used in phytoremediation as chelating and plant restoration agents. Citric and glutaric acids enhanced the accumulation of Cd and Pb in this experiment. The increase in Cd/Pb uptake showed an increasing trend with citric acid concentrations, whereas different concentrations of GA showed variations in accumulation. Similar observations were also reported in previous studies. OAs addition was effective for Cd absorption capacity of plants, even at high concentration [35]. Farid et al. [2] reported that the application of CA elevated the Pb accumulation by up to 48% in sunflower compared to respective controls. 

Generally, OAs can improve the solubility and bioavailability of heavy metals through promoting desorption of metals by the formation of soluble complexes with metal ions, thus increasing metal accumulation by plants [36]. Moreover, it was proven that carboxyl groups in OAs could reduce the biotoxicity of heavy metals in plants by chelation [37]. However, the performance of OAs on phytoextraction depended on various factors, such as joint toxicity of metals, chemical structures of organic acids and organo-metals, and physiological characteristics of plants [38]. In our experiment, GAs in some treatments inhibited the phytoextraction of Cd/Pb. This might be due to the stable formation of multi-ligand structure among Cd, Pb and GAs, which make bioavailability of Cd or Pb decrease, or augment toxicity to plants.

Moreover, the existence of Cd/Pb ions inhibited the accumulations of Pb/Cd in our study. This finding is in agreement with Guo et al. [21] and Niu et al. [39], who reported that 5 and 10 mg·kg^−1^ Cd increased the bioavailability of Pb ions in soil. Besides, studies have shown that the interaction of cadmium with lead or zinc at equimolar concentrations can lower the toxicity of cadmium in plants [40].

### 4.3. The Translocation of Cadmium and Lead in Sunflower

The value of the translocation factor (TF) is one of the important indices in screening hyperaccumulators of heavy metals [41]. The application of exogenous organic acids is helpful in augmenting Cd, Pb, and Cu concentrations in shoots of the plants, which is manifested by an increase in TF values. Wang et al. [42] found that CA could increase Cd transfer in tall fescue by formation of water-soluble organo-Cd complexes, and CA further raised the Pb concentration up to 15–22% in leaves, compared to only-Pb treated plants. Generally, stable chelating compounds, which formed from negatively-charged carboxyl or hydroxyl groups in OAs and positively-charged heavy metals, are conducive to the increasing concentrations of metals in plant shoots [11]. However, in our experiment, CA and GA had no effect, or hindered the translocation of Cd or Pb in some treatments. These results were consistent with the studies of Ashraf et al. [43] and Shi et al. [44], who found that some toxic metals (such as cadmium, zinc, lead and copper) chelated with OAs might cause a significant reduction in the movement of metals from roots to shoots.

### 4.4. Relations between OA Concentrations and PRB and Cd/Pb Uptake

In this experiment, Cd/Pb uptake by roots showed a positive trend with increasing concentrations of OA. The results were consistent with previous studies [45,46,47], which deemed that plants could adsorb more heavy metals with their increment within an appropriate range. 

PRBs and the amount of Cd/Pb in the plants were positively associated with the concentrations of CA, and these results were in accordance with the study of Jagetiya et al. [48], who described that, the higher the optimum amount of a chelating agent, the higher the biomass production, as CA, being a small molecule organic acid, could have been less toxic to the plant, and normalize the toxic organic pollutants in terms of their toxicity to plants. On the contrary, Wang et al. [49] considered that CA had no marked influence on plant growth, while it only showed a slight stimulatory effect at a concentration of 5 mmol·L^−1^. Wang et al. [50,51,52] indicated that the addition of CA apparently weakened Cd phytoextraction ability, which was mainly due to the chemical and structural properties of CA, and metal-chelator and ligand-metal ions complexes formed with carboxyl groups in CA would indirectly lead to Cd insolubility.

GA concentrations did not influence the uptake of Cd/Pb and PRB values, and they showed a negative effect on the uptake of metals in complex treatments. These observations were similar to the findings of Quartacci et al. [53], which indicated that the addition of chelating agents could result in an increased amount of heavy metal ions in the soil solution which would cause plant growth inhibition and biomass reduction. In most instances, when heavy metal availability is low, the activation rates of heavy metal ions increase initially and then plateau with the increase of OA concentrations [36,38]. The activation enhancement might lead to the toxicity being elevated to a more significant extent, which could attenuate the basis of the growth of plants and accumulation potential. 

In this study, neither concentration of OAs showed correlations with TF values, whereas the increments of CA and GA promoted the TF values of Cd/Pb individually in some treatments. These results were consistent with those of other researchers, who found that higher dosage of chelating agent would cause greater transportation of uranium and nickel in plants [41,48,54]. Schwab et al. [7] also found that Pb movement could be enhanced by higher concentrations of some OAs. On the other hand, Yanai et al. [55] considered that too high concentrations of some LWMOAs were unsuitable for the translocation of heavy metals, though the addition of appropriate concentrations could attenuate the peroxidation damage of the cytoplasmic membrane. The relationships between the dosage of OAs applied and the translocation of heavy metals need to be further studied, and are closely linked with the chemical structures of OAs, characteristics of complex, metals sequestration in roots, and so on.

## 5. Conclusions

Our research revealed that the addition of citric and glutaric acids could improve the growth of sunflowers in single Cd/Pb treatments, and the biomass increment of shoots and roots showed an uptrend with increasing concentrations of these two organic acids. In complex treatments, CAs could enhance the phytoextraction of cadmium and lead, but the addition of GAs showed inhibitory action on the uptake of metals. The application of both acids affected the translocation of Cd and Pb from roots to shoots differently, but there were no correlations between TFs and concentrations of OAs. On the whole, the amendment with citric and glutaric acids could be used to stimulate the phytoextraction of Cd and Pb by sunflower plants, but more research is needed to verify the effectiveness of OAs in phytoremediation.

## Data Availability

The data that support the findings of this study are available from the corresponding author upon reasonable request.
